# Evaluation of the impact of construction products on the environment by leaching of possibly hazardous substances

**DOI:** 10.1186/s12302-018-0144-2

**Published:** 2018-05-15

**Authors:** Nicole Bandow, Stefan Gartiser, Outi Ilvonen, Ute Schoknecht

**Affiliations:** 1 Bundesanstalt für Materialforschung und -prüfung, Unter den Eichen 87, 12205 Berlin, Germany; 2 Hydrotox GmbH, Bötzinger Straße 29, 79111 Freiburg, Germany; 3 Umweltbundesamt, Wörlitzer Platz 1, 06844 Dessau-Roßlau, Germany

**Keywords:** Prospective risk assessment, Groundwater, Surface water, Soil, Ecotoxicological tests, Targeted and non-targeted analysis, Construction products

## Abstract

Construction products are in contact with water (e.g., rain, seepage water) during their service lifetime and may release potentially harmful compounds by leaching processes. Monitoring studies showed that compounds attributed to construction products are found in storm water and the receiving bodies of water and that the release of biocides in urban areas can be comparable to the input of pesticides from agricultural uses. Therefore, a prospective risk assessment of such products is necessary. Laboratory leaching tests have been developed by the Technical Committee CEN/TC 351 and are ready to use. One major task in the future will be the evaluation of the leaching test results, as concentrations found in laboratory experiments are not directly comparable to the field situations. Another task will be the selection of compounds to be considered for construction products, which are often a complex mixture and contain additives, pigments, stabilization agents, etc. The formulations of the products may serve as a starting point, but total content is a poor predictor for leachability, and analysis of the eluates is necessary. In some cases, non-targeted approaches might be required to identify compounds in the eluates. In the identification process, plausibility checks referring to available information should be included. Ecotoxicological tests are a complementary method to test eluates, and the combined effects of all compounds—including degradation products—are included. A bio test battery has been applied in a round robin test and was published in a guidance document. Published studies on the ecotoxicity of construction products show the tests’ suitability to distinguish between products with small and larger effects on the environment.

## Introduction

Public awareness of the importance of the good quality of surface water developed in the 1970s when pollution, for example, of the Rhine River, was observable in foam, chemical smell, and major fish kills caused by an accident in the chemical industry in 1986 in Schweizerhalle [1]. Due to regulations on the discharge of wastewater, concentrations of many hazardous compounds showed a decreasing trend and surface water quality has improved. However, the monitoring performed for the European Water Framework Directive shows also that many bodies of water still do not have a good ecological status and that authorities are facing problems improving water quality sufficiently. The decreased impact of chemical industry and other industrial sectors, like the pulp and paper industry and the textile industry, on water quality is accompanied by a general shift from a restricted number of point sources to a more diffuse input from many different smaller sources. The increasing number of sources leads also to an increasingly necessary effort to identify sources. These sources include agriculture, wastewater treatment plants, atmospheric deposition, and urban run-off. While agricultural pesticide and fertilizer entry by run-off and drift has been in the focus of research for 40 years, e.g., [2, 3] and is already part of the authorization process for products, other sources like wastewater treatment plants came into focus only more recently, leading to the upgrading of tertiary sewage treatments for the removal of emerging pollutants.

Buildings and the applied construction materials (e.g., renders, mortars, paints, roofing materials, and bricks), as important factors for water quality in urban areas, are a newer field of research. Bucheli et al. [4] showed 20 years ago that high mecoprop concentrations in the run-off from roofs originate from the hydrolysis of a bi-ester of mecoprop used as an agent to protect bitumen sheets from root penetration; they estimated that the load in the receiving water bodies from roofs and agricultural applications were in the same order of magnitude. Mass flow analysis revealed that up to 50–80% of the load of heavy metals such as Cd, Cu, Pb, and Zn in combined sewer systems could be attributed to the run-off from roofs and streets [5].

Up to now, several monitoring studies of storm water have been conducted: biocides such as carbendazim, terbutryn, and diuron, as well as mecoprop, the transformation product of roof-protecting mecoprop esters, were found in a study of storm water from an urban catchment near Zurich [6]. A study of storm water in a catchment in the UK with an equivalent of 50,000 inhabitants proved the presence of polyaromatic hydrocarbons (PAH), trifluralin, nonylphenols, nonylphenol ethoxylates (NPEOs), and diethylhexyl phthalate (DEHP) [7]. Phthalates, nonylphenol, and NPEOs were also detected in storm water in two urban residential areas in Stockholm [8]. A study in the region of Paris investigated compounds in separate sewer systems and detected heavy metals (Cu, Zn), PAH, diuron, monobutyltin (MBT), DEHP, and nonylphenols as contaminants [9]. Storm water monitoring in Copenhagen demonstrated the presence of heavy metals such as Pb, Cd, Cr, Cu, Ni, and Zn, as well as PAH, diuron, isoproturon, glyphosate and its degradation product aminomethylphosphonic acid (AMPA), DEHP, and nonylphenol [10]. Xiao et al. [11] searched especially for perfluoroalkyl acids (PFAA) and were able to detect them in all storm water samples taken in the Minneapolis-St. Paul metropolitan area.

Contaminants in urban storm water can originate from various sources, such as parks/gardens, roads, and sealed surfaces [12], that are not exclusively linked to construction products. This includes diuron and glyphosate, which are also used in residential areas [13]. The situation is even more complicated if combined sewer systems are sampled. Elaborated strategies are necessary to distinguish between run-off and other sources. Gasperi et al. [14] compared the concentrations in separate and combined sewer systems and concluded that higher concentrations of PAHs and alkylphenol ethoxylates in the combined waste water indicate other sources than run-off. Wittmer et al. [15] conducted a comprehensive field study on the catchment scale and were able to assign concentration patterns to land uses. Bollmann et al. [16] used the different emission patterns of substances under dry and wet conditions to identify further sources, such as household chemicals. Rainwater can be collected to quantify the total atmospheric fallout in the catchment area under consideration [17]. Wicke et al. [18] were able to show that different types of construction areas, like areas of single-family homes, either old or new apartment buildings, industrial parks, and traffic areas result in different contamination patterns of storm water, which could be linked to possible usage of the chemicals.

The presence in the environment of chemicals originating from construction products underlines the need for a prospective assessment of such products. The aim of this publication is to give an overview of the current methodologies for assessing the release of potentially hazardous substances from construction products by contact with water (e.g., rain or seepage water). The emission to air is not covered. Special focus is laid on different strategies that can be applied to use leaching test data for regulation processes and on the gaps, that should be filled in the future to have a profound base of knowledge for decision-making.

## Regulatory context and leaching tests

In the European Union, it is commonly accepted that requirements for construction works include proof that they will not have adverse effects on human health and the environment. Therefore, “hygiene, health, and the environment” is a category of the basic requirements for construction works in European construction products regulation [19]. Constructions impact local environments during service time mainly due to emissions of substances into environmental compartments, such as the release of dangerous substances into groundwater, marine waters, surface waters, or soil. Expected emissions need to be estimated for construction products that can be in contact with water during their service life. The Technical Committee, CEN TC 351 “construction products: assessment of release of dangerous substances”, developed two laboratory test procedures to determine the amount of substances released into water. These horizontal leaching tests, as they are called, are intended to be generally applied for construction products with only a few exceptions. Where no additional information on the eluent is given, all standardized leaching tests presented here use demineralized water.

One of the tests—CEN/TS 16637-2:2014 [20]—was developed to investigate area-related leaching from the surfaces of monolithic construction products. This test is known as the dynamic surface leaching test (DSLT) and intends to describe diffusion-controlled leaching processes. Test specimens are exposed to water at a defined ratio of water volume to exposed surface area under controlled laboratory conditions. The water is exchanged on a defined time schedule and analyzed for the target substances. The contact time during the leaching intervals increases from 6 h at the beginning to 28 days at the end of the overall 64 days of test duration. Emissions per surface area are calculated from the obtained substance concentrations in the eluates and can be related to the duration of exposure. Based on the resulting emission curves, the test results make it possible to determine whether leaching is controlled by diffusion or other processes such as washout effects at the beginning. Diffusion-controlled processes are proportional to the square root of contact time and are indicated by the pattern of the eluate concentrations after the defined periods of water contact.

The test results cannot be used directly to derive expected environmental concentrations. Concepts for transferring results obtained under laboratory exposure conditions to service-life conditions still need to be developed or refined [21, 22]. This restriction applies especially to organic substances that can be transformed during service life or in water. However, the test indicates whether target substances can be leached from investigated construction products. It is also possible to compare the leachability of the target substances from different construction products. Duester et al. [23] developed a framework to evaluate the results from the DSLT test for amour stones in hydraulic engineering by defining maximum release limits (MRL). They included common use scenarios to calculate MRLs that meet concentrations of environmental standards for specified times without water exchange.

The other test—CEN/TS 16637-3:2016 [24]—is a percolation test that applies to mass-related release from granular construction products. Columns are filled with granular construction materials and exposed to up-flow percolation. The eluted water is collected stepwise until certain liquid-to-solid ratios are reached and can be analyzed for the target substances. The test results are presented as a function of liquid-to-solid ratios and make it possible to distinguish between different leaching mechanisms.

It is important to notice that the release in the column percolation test is mass-related, while it is area-related in the DSLT. European test laboratories [48] checked both test methods for robustness to certain test parameters in a comprehensive study of numerous representative construction products. Until now, only a few studies using these two tests have been published: Nebel and Spanka [25] showed the robustness of the DSLT for release from concrete. Hartwich and Vollpracht [26] applied the DSLT to determine the leaching behavior of concrete, and Märkl et al. [27] investigated the release of super-plasticizers from cement.

Another leaching test often applied especially for waste samples is the “one-stage batch test” in accordance with DIN EN 12457-1. In the technical specification CEN/TS 16637-1 of CEN/TC 351, this test is mentioned as an “indirect method”. Gartiser et al. [28, 29] have used this procedure to prepare leachates from granular construction products. Construction products exposed to weathering conditions with wet and dry phases can also be tested in a tank test with intermittent contact with water (immersion cycles) in accordance with DIN EN 16105, which was developed for paints and varnishes. This approach has been applied, e.g., to assess reactive fire-protecting coatings [30].

Kobetičová et al. [31] also suggest the preparation of water-accommodated fractions (WAFS) in accordance with OECD Guidance No. 23 on ecotoxicity testing of difficult substances and mixtures [32]. Here, defined amounts of the test item are directly weighted to the test-specific dilution water, stirred for about 24–72 h, and eluates are obtained after the separation of solids by sedimentation, filtration, or centrifugation.

It should be noted that biocidal products, such as wood preservatives and film preservatives in facade coatings, are submitted to a detailed environmental risk assessment under the Biocidal Products Regulation (EU) No 528/2012. Laboratory leaching tests for biocidal products that are based either on constant or intermittent water contact, as well as semi-field tests, are listed in a guidance document of the European Chemicals Agency, ECHA. These tests also apply to biocidal products that are included in construction products [33].

However, it is helpful to limit the number of tests run to facilitate comparability between tested products on the market. The horizontal leaching tests developed by CEN/TC 351 are intended to be generally applied for construction products with only a few exceptions. It will be the task of CEN Technical Committees that are responsible for the different harmonized construction product standards to select the applicable leaching test and define substances that are to be investigated. These results will be the basis for declaring the properties of construction products as part of CE marking for marketing within Europe.

This is a challenging task, since a reasonable selection of target substances requires detailed knowledge of the composition of construction products that can be the producers’ proprietary know-how. In addition, the leaching test conditions, especially water quality and the duration of water contact, do not necessarily reproduce service-life conditions. For instance, Hartwich and Vollpracht [26] observed that the leaching of heavy metals from concrete can be considerably reduced by the presence of calcium in leaching water that originates from natural sources, as compared with deionized water. This is explained by a precipitated layer of calcium carbonate on concrete surfaces that was also observed under service-life conditions. In general, the use of deionized water often leads to an alteration of the concentration gradient between the sample and the eluent in comparison with the field situation and, subsequently, with a change in leaching behavior. Deionized water has no buffering capacity and is prone to changes in pH value if products with high alkaline or acidic potential are investigated. The pH value is, therefore, an important parameter for the interpretation of leaching test results. The standardized test conditions of the harmonized leaching tests are, therefore, not to be considered a simulation of reality, but as a helpful convention that allows further modeling of the data. Another challenge is the interpretation of test results from leaching tests under constant water contact for construction products on structures above the ground. These include roofs and facades, which are only occasionally exposed to water contact. In the case of facades, it is also important to consider that these vertically oriented surfaces are exposed only to driving rain. It can be useful to apply a test procedure that is based on intermittent water contact such as EN 16105 [34], which was developed for paints and varnishes, depending on the properties and exposure conditions of the construction materials.

Analyses of eluates from construction products offer an excellent chance to avoid undesirable impacts on the environment. However, their benefit is directly linked to the selection of the investigated target substances. Candidate substances are summarized in the “indicative list” that shows inorganic and organic parameters that are specifically regulated for construction products in at least one of the European Union member states. Substances that need to be controlled can be selected from this list based on knowledge of the general composition of construction products. A working group from CEN TC 351 (WG 5) is currently harmonizing analytic methods for the quantification of these substances in eluates. Nevertheless, due to the large variety of materials used in construction, this list can cover only some of the substances that can have environmental impacts. This makes it possible not only to assess the release of selected compounds, but also to identify compounds of interest by non-targeted analysis and to assess the effects that leachates have on organisms. Both approaches will be discussed in the following paragraphs.

## Chemical analytical methods

The published reports on studies dealing with the release of compounds from construction products and the monitoring of compounds in storm water usually analyze a limited number of analytes. The release of biocides (e.g., [35–39]) and metalloids [40–43] has been intensively studied, while only a few studies have dealt with selected other compounds, like the release of bisphenol A from corrosion protection products [44], the release of thiocyanate and nonylphenol from concrete [45], the release of DEHP and selected alternative plasticizers from PVC [46], and the release of PAH and phenols from bitumen used to waterproof roofs [47]. The robustness study performed for the leaching standards for construction products included inorganic analytes for a broad range of products and also considered the release of PAH, biocides, and alkylphenols from different products [48]. Monitoring studies often focus on the list of priority substances as defined in the EU water framework directive [7, 9, 10].

Construction products are usually complex mixtures containing many more chemicals than included on the list of priority substances: residues from the production process and additives such as plasticizers, stabilization agents, pigments, surfactants, and solvents. In addition, the transformation of substances during the service life can result in as yet unknown substances and will be an important task for non-targeted analysis and fundamental research. Identifying relevant substances leaching from the materials is, therefore, quite a challenge. Usually, the process is a stepwise approach and depends on the information available. The formulation of the product, if it is available, is a good starting point for substance identification. This compound list can be checked for substances that are classified as environmental hazards in accordance with the globally harmonized system of classifying and labeling chemicals [49] or those included in the indicative list [50]. Especially substances with a high acute toxicity (hazard statement H400) and with long-lasting effects (H410, H411) are of concern. Knowledge of the presence of potential dangerous substances alone is not sufficient information, as total content is a poor indicator of the leaching potential and the resulting concentrations in the eluates. Furthermore, dangerous substances in the bulk material do not have to be reported if the concentrations are below reporting thresholds.

The screening of eluates is another way to identify relevant substances. While for inorganic analytes, the number of possible analytes is limited (if the analysis of different species of a single element is not considered), the number of organic compounds including degradation products is very high. Either gas chromatography coupled with mass-selective detection (GC–MS) or liquid chromatography coupled with mass-selective detection (LC–MS) might be used for screening. GC–MS with electron ionization (EI) at a given ionization energy of 70 eV provides robust and reproducible fragmentation of compounds, allowing the comparison of mass spectra even if they were recorded using different types of instruments. The availability of commercial mass spectra libraries facilitates identification. However, this approach is limited by the number of GC–EI–MS spectra of compounds stored in the libraries (NIST 262,000 compounds and Wiley 600,000 compounds), which is limited in comparison with the more than 135 million inorganic and organic chemicals included in the CAS registry. The structural elucidation of compounds not included in the libraries is hampered by hard ionization techniques, which often do not allow the identification of the molecular ion [51], and by the sometimes high number of molecular formulas that can be attributed to low-resolution mass spectra [52]. Even with more sophisticated methods, such as the use of MS classifiers [52] and fragmentation patterns [53], structural elucidation is challenging. Furthermore, GC–MS is limited to volatile compounds, and the sample preparation steps necessary to transfer analytes from aqueous eluates to an organic solvent compatible with GC–MS may bias identification of certain analytes. In addition, sample preparation procedures do not necessarily select between analytes and other substances. Therefore, a concentration of additional substances in the eluates can increase disturbing matrix effects on quantification methods.

Sample preparation with the aforementioned drawbacks may also be used for LC to enrich the analytes by e.g., solid phase extraction. The selection of the chromatographic conditions and the ionization technique, such as electrospray ionization (ESI), atmospheric pressure chemical ionization (APCI), or atmospheric pressure photoionization (APPI), and the use of positive and/or negative mode can miss certain analytes [54]. The results of a collaborative trial for non-targeted screenings organized by the NORMAN Association (network of reference laboratories, research centers, and related organizations for monitoring of emerging environmental substances) reveal that generic HPLC methods using C_18_-columns are suited; but especially for very polar compounds with early retention times, special methods may have to be used [55]. The absence of comprehensive spectra libraries necessitates an elaborated strategy for data handling. This automated data processing includes subtracting signals present in blank samples, filtering instrumental noise, mass filtering, peak detection, and the search for homologue series. High mass accuracies together with isotopic structures are essential for assigning a molecular formula, which can be used to search databases such as Pubchem and Scifinder for possible chemical structures. These structures are used to predict fragmentation and retention times in silico and to rank the candidates by the match factors of prediction and measured data [51, 56]. This approach is limited to compounds present in databases. Structure elucidation from the experimental data alone is very challenging; Holcapek et al. [54] have compiled characteristic fragmentation behavior for small molecules.

Not all compounds tentatively identified by the exact mass of adducts of the molecular ion or the fragmentation pattern can be further considered, and plausibility should be evaluated using all available data. The ECHA chemical database might be helpful to identify chemicals with high production and use volume. Furthermore, information on typical usage is given. Environmental monitoring data can also help to identify relevant substances. However, it should be noted that lack of environmental data does not necessarily mean that these compounds are not present, if they were not included in monitoring programs. Selected identified compounds have to be fully confirmed by means of analytical standards as reference.

Chemical monitoring alone can never be complete, as the sampling and sample preparation strategy already miss certain analytes. This is also true of the detection method. Furthermore, in non-targeted screening approaches, only some of the compounds present are usually identified. Identification of every small signal near the limit of detection seems impossible. Chemical concentrations alone can be linked to the ecological relevance of these compounds only if information on possible negative effects is available. Assessing the effects caused by the leachate might, therefore, be a tool supplementary to chemical analysis.

## Ecotoxicity tests

Ecotoxicity tests can be a valuable tool to indicate whether environmental impact has to be expected from a certain construction product. This is especially important either if comprehensive chemical analysis of eluates is not possible, or if the environmental impact of the analyzed substances is unknown. While the ecotoxicity testing of waste is generally accepted and included by Regulation (EC) 2017/997 in Waste Directive 2008/98/EC for determining the hazardous property HP 14 “ecotoxic waste”, few studies have considered the direct ecotoxicity testing of construction products so far [57].

In a recent review, Kobetičová and Černý [31] described several approaches to life-cycle ecotoxicological assessment of building products. The authors started with the impact of mining or quarrying raw material on soil fauna and of the manufacture of cement, concrete, and ceramics. They state that ecotoxicity studies of building materials “in situ”, i.e., in the construction phase or during their service life, are very rare. These studies also cover borderline areas not specifically attributed to construction products, such as coatings containing biocidal film preservatives or nano-silver. None of the studies referred to deals with the pre-marketing assessment of a formulated construction product.

Similarly, Rodrigues et al. [58] proposed an evaluation scheme to assess virgin and recycled raw materials and construction materials, based mainly on a French proposal for classifying waste. The proposal includes the one-stage batch test in accordance with EN 12457-4 (L/S ratio 10 L/kg) and chemical and ecotoxicological testing using luminescent bacteria, the *Daphnia magna* and the *Saccharomyces cerevisiae* growth tests.

More often, the known ingredients of a construction product are analyzed in leachates and compared with water quality standards [37] or known ecotoxicity data [59] to assess environmental hazards. This approach, however, requires knowledge of the ingredients in construction products and/or extensive chemical analysis. The combined effects of all leachable substances and transformation products are determined by their total effects in bioassays.

In the field of construction products, several studies considered the leaching or run-off of biocides or metals from outdoor structures. Some of these studies have been combined with ecotoxicity tests. Heijerick et al. [60] analyzed the bioavailable zinc concentration in rain-driven run-off from zinc-coated panels (300 cm^2^) and explained the observed effects on genetically modified luminescent bacteria *Alcaligenes eutrophus* and algae *Raphidocelis subcapitata* in the presence of Zn^2+^ ions.

In only a few studies, eluates from leaching tests have been combined with ecotoxicity tests (see Table 1). It should be noted that observed ecotoxicity does not necessarily mean that negative effects are observed also in the environment. The results of the different studies can hardly be compared, because different leaching tests were used and the reported endpoints vary. The results strongly depend on parameters of the leaching test, like the water volume used for leaching. Furthermore, the choice of eluate fractions has a strong influence: Heisterkamp et al. found higher toxicity for fraction seven of a DLST in comparison to the combined first two eluate fractions. This was explained by the later fraction’s longer contact time in the leaching phase and by the swelling of the coatings. Sudár et al. [61] used different dilutions of the eluates for ecotoxicity testing and established dose–response curves. These curves were used to calculate the eluate dilution where 50% of the effect is observed (EC_50_). They concluded that no direct adverse effects on aquatic ecosystems are expected if only eluates with a maximum dilution of 1:1 cause 50% of the effect. Comparative analysis with leachates obtained from column tests, which are suggested to better reproduce field conditions, generally showed lower or no ecotoxic effects, compared with eluates obtained from batch tests [62].

**Table 1:** Studies combining leaching tests and bioassays

Sample type	Leaching test fraction for bioassay water quality	Test species and standard for bioassays	Results^a^	Literature source
Two PAC (waterproof layer)	Column percolation Fraction for bioassays selected according to total organic carbon content Drinking water	*Vibrio fischeri* DIN ISO 11348-2	No toxicity observed	Wagner [63]
		*Desmodesmus subspicatus* DIN 38412-33	PAC1: LID > 16 PAC2: LID > 1024	
PVC/PU floor	EN 12457-4 One-stage batch L/S 10 L/kg Deionized water	*Daphnia magna* ISO 6341:1996	EC_50_, _24 h_ = 71 g/L EC_50_, _48 h_ = 52 g/L	Lithner et al. [74]
HDPE, MDPE, ABS, PVC, PP water pipes	Batch test 72 h, L/S 6–15 L/kg Deionized water	*Daphnia magna* ISO 6341:1996	No toxicity observed	Lithner et al. [74]
Recycled polypropylene/wood composites	EN 12457-1 One-stage batch Deionized water	*Vibrio fischeri*	DF_50_ > 2 (i.e., 50% eluate in test samples)	Sudár et al. [61]
Four anti-corrosion coatings based on epoxy resins Coatings were cured before testing for 1 or 7 days	Non-standardized tank test, continuous turbulence of the eluent Ultrapure water	*Aliivibrio fischeri* bioluminescence, Microtox assay	Product 1: LID_1 day_ 1282; LID_7 days_ 171 Product 2: LID_1 day_ < 2; LID_7 days_ 2 Product 3: LID_1 day_ 4; LID_7 days_ 8 Product 4: no toxicity observed	Vermeirssen et al. [44]
		*Pseudokirchnerialla subcapitata* Photosynthesis, growth rate	Product 1: no toxicity observed Product 2: no toxicity observed Product 3: LID_1 day_ 4; LID_7 days_ 10 Product 4: no toxicity observed	
		*Ceriodaphnia dubia* ISO/CD 20665 and AFNOR T90-376	Product 1: LID_1 day_ 5; LID_7 days_ 8 Product 2: LID_1 day_ 2; LID_7 days_ 22 Product 3: LID_1 day_ 10; LID_7 days_ 67 Product 4: LID_1 day_ 3; LID_7 days_ 2	
Organic render containing either free or encapsulated biocides (terbutryn, OIT, DCOIT) compared to render without biocides	EN 16105 (intermittent water contact), eluates from 1st and 9th immersion cycle Deionized water	*Pseudokirchneriella subcapitata* Photosynthesis, growth rate	Free: 1st: EC_50_ = 630 9th: EC_50_ = 120 Encapsulated: 1st: EC_50_ = 130 9th: EC_50_ = 30	Burkhardt et al. [75]
		*Aliivibrio fischeri* bioluminescence	Free: 1st: EC_50_ = 58 9th: EC_50_ = 4.6 Encapsulated: 1st: EC_50_ = 5.0 9th: no toxicity observed	
		*Ceriodaphnia dubia* ISO/CD 20665 and AFNOR T90-376	Free: 1st: EC_50_ = 21 9th: EC_50_ = 7.0 Encapsulated: 1st: EC_50_ = 6.5 9th: no toxicity observed	
		*Eisenia fetida* ISO 17512-1	No toxicity observed	
		*Folsomia fimetaria* OECD 232	No toxicity observed	
Three reactive flame-proof coatings	CEN/TS 16337-2 DSLT Fraction 1 + 2 Fraction 7 Deionized water	*Danio rerio* DIN EN ISO 15088	No toxicity observed	Heisterkamp et al. [30]
		*umu*-*Test* ISO 13829	No genotoxicity observed	
		*Pseudokirchneriella subcapitata* ISO 8692	Fraction 1 + 2: P1: LID = 16; P2: LID = 2, P3: LID = 3 Fraction 7: P1: LID = 32; P2: LID = 1, P3: LID = 8	
		*Daphnia magna* DIN EN ISO 6341	Fraction 7: P1: LID = 6 Other fractions: no toxicity observed	
		*Vibrio fischeri* DIN EN ISO 11348-2	Fraction 1 + 2: P1: LID = 24, P2 and P3 no toxicity Fraction 7: P1: LID = 256, P2: LID = 16, P3: LID = 12	
EPDM SBR EL	E DIN 1957/DIN 19529 Batch test L/S = 2 L/kg Ultrapure water	*Pseudokirchneriella subcapitata* ISO 8692	EPDM, SBR, EL: LID = 33	Krüger et al. [62]
		*Daphnia magna* ISO 6341	EPDM: LID > 243; SBR: LID = 27; El: LID > 28	
Four complete artificial turf systems	DIN 19528 column percolation test Ultrapure water	*Pseudokirchneriella subcapitata* ISO 8692	P1 and P3: LID = 10 P2 and P4: LID = 3	Krüger et al. [62]
		*Daphnia magna* ISO 6341	P1 and P4: LID = 9 P2: LID = 27 P3: no toxicity observed	

*PAC* polyacrylate, *PVC* polyvinyl chloride, *PU* polyurethane, *HDPE* high-density polyethylene, *MDPE* medium-density polyethylene, *ABS* acrylonitrile butadiene styrene, *PP* polypropylene, *OIT* 2-octyl-3(2H)-isothiazolinone, *DCOIT* 4,5-dichloro-2-*n*-octyl-4-isothiazolino-3-one, *EPDM* ethylene propylene diene monomer rubber, *SBR* styrene butadiene rubber, *EL* elastic layer

^a^*EC*_*50*_ concentration, that causes 50% effect, *LID* lowest ineffective dilution, *DF*_*50*_ eluate dilution that causes 50% effect eluate is diluted to obtain dose response curves, DF_50_ is derived from this curve

Wagner [63] did not expect long-term impacts because the substances were degraded in the screening test in accordance with OECD 301 E [biodegradability by change in concentration of dissolved organic carbon (DOC)]. In additional terrestrial tests, the inhibition of nitrification was tested in accordance with the ammonium oxidation test (ISO 15685) and the soil microflora abundance and activity test, using respiration curves (ISO 17155). While in the 1st week, an inhibition of nitrification of up to 28% was observed, the respiration of soil microflora increased. Terytze et al. [64, 65] showed that the nitrification inhibition and soil microflora respiration tests are sensitive enough to indicate negative effects of construction materials on soil organisms.

Gartiser et al. [28, 29] investigated the suitability of harmonized test methods to describe ecotoxic effects of eluates from construction products. Their study examined aquatic test methods that had already been proposed to evaluate waste [66, 67] and ecotoxicity tests that are applied to assess construction products in Germany [68, 69] for their suitability to investigate eluates from construction products. Leaching experiments were performed on twenty construction products of different origin and composition. Monolithic and plate-like products were eluted in accordance with CEN/TS 16637-2 [20], whereas granular products were eluted in a one-stage batch test in accordance with EN 12457-1 [70]. The eluates were examined in four aquatic toxicity tests (algae, daphnia, luminescent bacteria, fish eggs), a genotoxicity test (umu test), and the respirometer test for biodegradability (OECD 301 F). In this test, the lowest ineffective dilution (LID) was determined. For the determination of the LID, the sample is diluted until the observed ecotoxicity dropped below a level at which no inhibitions, or only effects not exceeding the test-specific variability, are observed (e.g., growth inhibition green algae < 5%). The observed ecotoxicity of these eluates differed considerably. Many of the twenty products tested showed no effects in ecotoxicity tests. However, there were also eluates from products such as ethylene propylene diene monomer rubber (EPDM), sealing masses, and synthetic flooring that had to be diluted up to a dilution factor above 1000 to derive LID values. For six out of eight investigated eluates that contained more than 10 mg/L TOC (total organic carbon), biodegradability was above 75% [29]. A round robin test including seventeen laboratories from five EU member states was performed using eluates from leaching tests on a monolithic and a granular construction material that were both based on EPDM. The selected ecotoxicity tests with algae, daphnia, and luminescent bacteria were able to demonstrate toxic effects, as well as the level of toxicity of the eluates. The eluates were not toxic to fish eggs. The reproducibility of the tests was evaluated based on effective concentration 50 (EC_50_) and lowest ineffective dilution (LID) values. It was observed that the variability of the test results increased with the overall level of toxicity and depended on the sensitivity of the test. Considering the complex overall process, the reproducibility of bioassays with eluates from construction products was regarded as acceptable [29]. Finally, a test battery for the ecotoxicological evaluation of the environmental safety of construction products was recommended [28].

Recently, a guidance document on the use of ecotoxicity tests applied to construction products was prepared in CEN/TC 351 and published in 2017 [71]. It refers to existing internationally harmonized ecotoxicity tests that were developed to assess the ecotoxic potential of chemicals, waste water, or contaminated soils and that more recently have been successfully applied to waste and waste eluates. It states that these methods, with some modifications, can be applied for the ecotoxicological characterization of construction products and their eluates.

The Guidance on the use of ecotoxicity tests for construction products, CEN/TR 17105, refers to the chemical-specific approach and a toxicity-based approach that complement each other. For complex mixtures of unknown or undisclosed composition, direct toxicity testing is recognized as a practicable method to estimate ecotoxicity. Ecotoxicity testing can be applied to construction product eluates (aquatic tests) or to construction products mixed with artificial soil (terrestrial tests). The selection depends on the intended use, i.e., whether it is exposed to soil or water. In the context of harmonized specifications for construction products, currently only Germany requests data on ecotoxicity/biodegradability in certain cases [68]. The DIBt concept for assessing the impact of construction works on soil and groundwater defines the following limit (rejection) values: LID > 4 for the algae and daphnia tests, LID > 6 for the fish egg test, and LID > 8 for the luminescent bacteria test. No genotoxicity is allowed in eluates. These tests are demanded, if ecological safety cannot be verified by evaluating ingredients, with leaching tests combined with chemical analysis, or by comparing measured concentrations with limit values. Thus, the added value of the ecotoxicity testing of construction products is mainly due to complex products with unknown or unassessed organic ingredients.

For the waste sector, which also concerns (used or recycled) construction products, more data on the direct ecotoxicity testing of eluates are available. Usually, a one-stage batch test with a liquid/solid ratio of 10 in accordance with EN 14735 [72] is prepared for subsequent ecotoxicity testing, and a limit (acceptance) value of LID ≤ 8 in the aquatic algae, daphnia, and luminescent bacteria test, as well as in the terrestrial tests with *Arthrobacter globiformis*, higher plants, and earthworms [73], is applied.

## Conclusions

Methods for determining the release of substances from construction products during contact with water have been developed in recent years. The standards are in the stage of validation by a round robin test and are ready to use. These leaching tests can be used to determine the leaching potential of certain compounds, but the concentrations are not directly comparable to concentrations measured in the field. Assessment of leaching test results requires not only transfer models of realistic exposure but also specification of acceptable environmental concentrations. One important task will be to decide which parameters should be considered and included in the analysis. As it will be impossible to identify all compounds in the eluates by means of chemical analysis, a complete assessment requires combining the analysis with ecotoxicity tests.

To receive reliable and comparable results, it is strongly recommended to apply only standardized ecotoxicity methods, giving preference to those referred to in the CEN/TR 17105 guidance. Later, when defining limit values, the different leaching conditions should carefully be considered. This is especially true for the water/area or water/solid ratios, which have a decisive influence on ecotoxicity and may differ in a wide range (from 20 to 80 L/m^2^ and 2 to 10 L/kg, respectively). For the DSLT, often the first two fractions are used for ecotoxicity testing, which may be influenced by wash-off peaks from surfaces and thus not be representative for assessing effects at later stages. Thus, another fraction from a later stage of the DSLT may additionally be tested. However, a long contact time may also have the disadvantage that (bio)degradable ingredients are not detected.

Despite the benefits of ecotoxicity tests, it must not be neglected that these tests can indicate only selected effects on organisms. The combination of tests on different organisms is based on important life processes but cannot be comprehensive. In addition, substances of concern cannot be identified by ecotoxicity tests. Chemical analysis is required to identify substances that cause ecotoxic effects. Both chemical analysis and ecotoxicity tests are needed to support the further development of environmentally friendly construction materials.
